# The global prevalence *ptxP3* lineage of *Bordetella pertussis* was rare in young children with the co-purified aPV vaccination: a 5 years retrospective study

**DOI:** 10.1186/s12879-020-05332-9

**Published:** 2020-08-19

**Authors:** Zengguo Wang, Yang Luan, Quanli Du, Chang Shu, Xiaokang Peng, Huijing Wei, Tiejun Hou, Ying Liu, Xiaoguai Liu, Yarong Li

**Affiliations:** 1 Xi’an Children’s Hospital, 69 Xijunyuan Road, Xi’an, 710002 Shaanxi Province China; 2 Xi’an Center for Disease Control and Prevention, 599 Xiying Road, Xi’an, 710054 China

**Keywords:** Bordetella pertussis, Pertussis, Acellular pertussis vaccine, Resistance, Membrane protein

## Abstract

**Background:**

The global prevalent *ptxP3* strains varies from about 10% to about 50% of circulating *B. pertussis* population in different areas of China.

**Methods:**

To investigate the difference of vaccination status between different genotypes in the circulating *B. pertussis* after 10 years of acellular pertussis vaccine (aPV) used in China. The nasopharyngeal swabs and isolates of *B. pertussis* from these patients were used to perform genotyping of antigen genes. We use antibiotic susceptibility test against erythromycin and sequencing methods for site 2047 of 23S rRNA to determine the resistance status.

**Results:**

The *ptxP1* allele with erythromycin resistant (ER) *B. pertussis* infection (total of 449 subjects) consisted of 84.70 to 96.70% from 2012 to 2016 in this study. Vaccinated with co-purified aPV was found in 133(133/403,33.0%), 1(1/9,11.1%) and 2(2/21,9.5%) in *ptxP1/fhaB3*-ER, *ptxP1/fhaB2*-ES and *ptxP3/fhaB2*-ES *B. pertussis* infected children each, which showed a significant difference (χ^2^ = 6.87, *P* = 0.032).

**Conclusions:**

The *ptxP3*-ES *B. pertussis* was rare while the *ptxP1-*ER *B. pertussis* was steadily increased in Xi’an, China from 2012 to 2016, where co-purified aPV was prevalent used. This pose a hypothesis that the co-purified aPV might protect against *ptxP3* strains more efficient, which generated a rare chance for *ptxP3* strains to be under the antibiotic pressure and further developed to be erythromycin resistance. A further cohort study and the mechanisms of the additional antigen proteins of co-purified aPV protected against *B. pertussis* should be consideration.

## Background

Pertussis is a respiratory disease mainly caused by *Bordetella pertussis*. The incidence of pertussis marked decreased after the whole cell pertussis vaccine (wPV) has introduced all over the world. Since the 1990s, a resurgence of pertussis has appeared in many countries, especially when the acellular pertussis vaccine (aPV) has replaced from the wPV. Furthermore, the circulating *B. Pertussis* has evolved mainly changed in the vaccine antigen genes proposed by the vaccine-driven, such as the *ptxP1* lineage to *ptxP3* lineage and also pertactin deficient [1]. Nowadays, the *ptxP3* lineage with/or without pertactin deficient strains, which has been proved to be more virulent and reflect selective advantage under the high coverage of aPV vaccination, has emerged globally and raised an important public issue toward an alternative vaccine in pertussis prevention [2].

However, except what happened in some countries like Iran, the *ptxP1* lineage was still prevalence in most countries used wPVs [3]. We have reported the *ptxP1* strains further shown erythromycin resistance (ER) that emerged in China since 2012. Furthermore, we found that all the *ptxP1-* ER strains originated from a *fhaB3* lineage, which appears to have been selected from the wPV or antibiotic pressure [4]. Interestingly, although the *ptxP1-*ER strains expanded all the countries of China, the proportions of *ptxP3-*ES strains varied from less than 10% to about 50% in different areas of China, especially occurred much higher in developed areas [5, 6].

The aPV came in two varieties according to the producing procedures: one is obtained through co-purified procedures so called co-purified aPV, which was used primarily in China and Japan. The other one with purification of each one to five components individually antigen and then blending them in an appropriate ration called purified aPV, which was used in lots of areas all over the world [7]. In China, the co-purified aPV was free and used predominantly since 2006. The purified aPV (Sanofi) was imported and rechargeable since 2011 and supplied much more in developed areas in China. Despite the *ptxP3*-ES strains were proved to be adapted to the purified aPV all over the world and also been prevalence under wPV vaccination in Iran [3, 8] . Moreover, whether the different proportions of *ptxP3*-ES strains in China were associated with the types of aPV remains enigmatic.

In this study, we conducted a 5-year retrospective study to survey the dynamic changes in genetic makeup & resistance status of the circulating *B. pertussis* and further the difference in demographic characteristics between different genotypes in Xi’an China, where co-purified aPV was still prevalence used. We are kind of hoping studies such as this can give more information in consideration of the modified vaccine for global pertussis prevention.

## Methods

### Study populations, strains and samples

All the patients admitted to Xi’an Children's Hospital for suspected of pertussis from 2012 to 2016 were sampled of nasopharyngeal swabs (NPs) and diagnostic by culture and special PCR for *B. pertussis* as previous reported [9, 10]. The DNAs from *B. pertussis* ATCC 9797 and ddH_2_O were used as positive and negative control in each PCR run [9]. The demographic characteristics were collected if culture and/or special PCR for *B. pertusssis* was positive. Totally, 702 cases were positive by the special PCR which including 204 cases with both culture and PCR positive of *B. pertussis*. All the strains and NPs were stored at − 80 °C until to use.

### Antibiotic susceptibility test

In-vitro sensitivity of clinical strains against erythromycin was performed and reported as previously [9]. We used *B. pertussis* ATCC 9797 and *Staphylococcus aureus* ATCC 25923 as controls.

### 23S rRNA sequencing and antigen gene typing

The nucleotide position 2047 of the 23S rRNA was performed by DNAs of strains and/or NPs by our previously reported sequencing methods [9]. Cause the A2047G of 23S rRNA was associated with erythromycin resistance, if the nucleotide position 2047 of the 23S rRNA was the wild type as adenine (A), we defined as an erythromycin sensitive *B. pertussis* infection. A mutation type as guanine (G) of site 2047 was taken for erythromycin resistance *B. pertussis* infection strain [11, 12]. The allele of *ptxP* and *fhaB* was performed by DNAs of strains and/or NPs as previously reported when successful sequencing of 23S rRNA [13].

### Statistical analysis

Data were statistically analyzed with SPSS 17.0. Comparisons were performed using χ^2^ test or one-way analysis of variance (ANOVA). A *P* value < 0.05 was considered statistical significant.

### Results

In total of 204 isolates, 3 strains were *ptxP3* allele and the others were all *ptxP1* alleles. Four strains, including of 3 *ptxP3/fhaB2* and 1 *ptxP1/fhaB2* allele, have the MICs against erythromycin lower than 0.023 μg/ml, which refers to sensitive to erythromycin in vitro. The rest of the 200 strains were all resistant to erythromycin with the MICs≥256 μg/ml, which were all *ptxP1/fhaB3* allele. All the resistant strains posed an A2047G mutation in 23S rRNA and no mutation occurred this site of the sensitive strains.

**Fig. 1:**
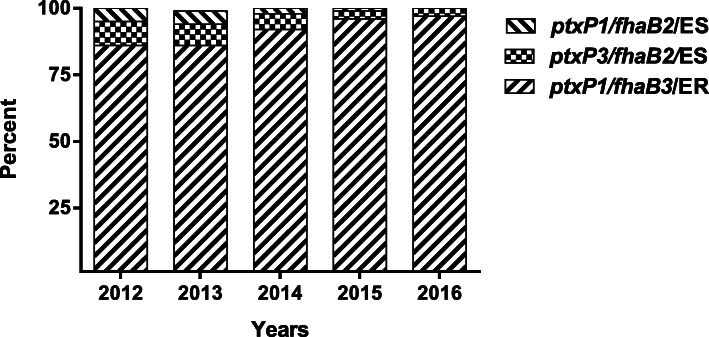
The proportions of different *B. pertussis* lineage in *B. pertussis* population from 2012 to 2016 in Xi’an, China

Among the 702 NPs for sequencing, 480 obtained both the available sequencing results of 23 rRNA, *ptxP* and *fhaB*. The NPs generated the sequencing results as same as obtained by strains from the same patient. There were 449 in 480 specimens (93.5%) shown the allele G in 2047 site of 23 rRNA that defined as erythromycin resistant *B. pertussis* infection, which also shown the allele of *ptxP1/fhaB3*. The dynamic changes of proportions of circulating *B. pertussis* from 2012 to 2016 as shown in Fig. 1.

Furthermore, 47 patients were excluded when analysis the difference among demographic characteristics cause of unclear vaccination status. Among the remaining 433 patients, 136 patients have been administrated with least 1 does of co-purifid aPV. Vaccinated with co-purified aPV was found in 133(133/403,33.0%), 1(1/9,11.1%) and 2(2/21,9.5%) in *ptxP1/fhaB3*-ER, *ptxP1/fhaB2*-ES and *ptxP3/fhaB2*-ES infected children, which showed a significant difference (χ^2^ = 6.87, *P* = 0.032). (Table 1).

**Table 1:** The demographic characteristics of children suffering from pertussis with different genetic makeup & erythromycin resistance status of *B. pertussis*

	*ptxP1/fhaB3-*ER	*ptxP3/fhaB2-*ES^a^	*ptxP1/fhaB2-*ES	χ^2^	P
	*n* = 403(%)	*n* = 21(%)	*n* = 9(%)		
Age (Months)^b^	3 (2–5.5)	2 (1–3.5)	3 (2–5)	1.479^c^	0.225
Vaccination status^d^				6.87	0.032
Vaccinated	133 (33.0)	2 (9.5)	1 (11.1)		
Unvaccinated	270 (67.0)	19 (90.5)	8 (88.9)		

^a^The *ptxP3*-ES with proportions of 8.93, 9.38, 6.19, 2.65 and 3.09% from 2012 to 2016 in this study

^b^The ages were represented as Med, x._5_ (Q1, x._25_- Q3, x._75_)

^c^Refers to the F value with ANOVA test between *ptxP1*-ER and *ptxP3*-ES group

^d^The cases of unclear vaccination status were not enrolled, what was 46 and 1 in *ptxP1*-ER and *ptxP3*-ES group each. All the vaccinated group administrated with the Co-aPV

## Discussion

Within our study, we discovered that *ptxP1*-ER strains have been steadily increased to the circulating *B. pertussis* population from 2012 to 2016 in Xi’an, China. Moreover, unlike that *B. pertussis* could not only infect the infants that were too young to be vaccinated, but also the infants vaccinated with the purified aPV [14, 15], the *ptxP3* strains rarely infected the infants administrated with co-purified aPV from our study.

The *ptxP3* strains have been circled predominant all over the world, especially after the replacement of wPV by aPV. However, the *ptxP3* strains have spread across the globe seems not only driven by aPV selection, but also by the fitness of *ptxP3* strains when compete with erythromycin sensitive non- *ptxP3* strains [3, 8]. Furthermore, we assumed that the quality of the wPV between different batch and/or the frequency of international movement of people possibly accelerated the prevalence of *ptxP3* strains in Iran too. The increasing incidence of pertussis was also emerged in China from 2013 according to the national infectious diseases case reported system. Besides the A2047G mutation in 23S rRNA occurred in *ptxP1-*ER *B. pertussis* strains, a novel *fhaB* C5330T was also founded in all these strains but didn’t appear in any *ptxP3* lineage. This *fhaB3* lineage has been proved to be prevalence among China via expansions most likely due to vaccine and/or antibiotic pressure [4]. This study illustrated that the *ptxP1/fhaB3-*ER strains might be adapted to the co-purified aPV more easily than global *ptxP3* strains.

It was reported that the *ptxP3* strains have been occurred in 2000 and remains sporadic in this country [16]. According to this study, *ptxP3* strains with the decreased proportions have observed from 2012 to 2016 in Xi’an, the western of China. In China, the co-purified aPV was free and predominated used since 2006 while the purified aPV (Sanofi) was available by paid since 2011with rarely market supplied, especially in undeveloped regions of western China, such as Xi’an. The rare of *ptxP3* strains in Xi’an after 10 years of co-purified aPV used might give a clue that the co-purified aPV did not give the adaption as purified aPV did in developed countries where *ptxP3* was quickly predominant worldwide [8]. The co-purified aPVs have more protein antigen than purified aPVs [17]. Therefore, this study further supported the hypothesis that the small antigen targets of purified aPV could induce the vaccine pressure and vaccine adaption more easily than the more antigen targets vaccine, such as wPV, even the co-purified aPV [18]. Furthermore, among the additional protein antigens of co-purified aPV, most was the out membrane proteins such as BipA and SphB1. Such membrane proteins containing in the outer membrane vesicles (OMVs) of *B. pertussis* have been suggested as an attracting candidate component of the possible new modified vaccine against pertussis [19, 20]. The latest study further proved that the OMVs can protect against *B. pertussis* with long term duration, even the global popular *ptxP3* and pertactin deficient strains [20]. Japan was the first country to develop aPV (co-purified) in 1981 and to adopt for use in the general population. It has been reported that both of the two types of aPVs was used recently [21]. However, the *ptxP3* lineage still holds lower than 50% from 2006 to 2010 until the period of 2011-2014which reached close to 80% [22].

Most of the cases in this study were from the west of China. Otherwise, it was reported that the *ptxP1*-ER strains contributed to 75.4, 50.7 and 48.6% in the circulating strains in Zhejiang province (Southern of China, 2016), Shanghai (Southern of China, 2016–2017) and Shenzhen (Southern of China,2015–2017), while the rest ES strains were almost *ptxP3* strains [5, 23, 24]. No details of the vaccine type were described in these relative high proportion of *ptxP3* areas of China. Liking what happened to Japan, we assumed that the purified aPV used was much more in these developed areas of China than in Xi’an, which generate a relative low level of vaccine protection from co-purified aPV in general population. As a result, the proportions of *ptxP3* strains were much more.

Consistent with reports in these areas of China, the erythromycin resistant strains were all *ptxP1* allele while the *ptxP3* strains were all sensitive to erythromycin. As shown in this study, though the average age of *ptxP3-*ES strains infection group is lower than in *ptxP1-*ER groups, no statistic significant difference was observed. Furthermore, more than 85% of subjects have taken antibiotics before sampling and detection, no difference was observed between the *ptxP3-* ES and *ptxP1-*ER groups (data not shown). Therefore, despite the antibiotic pressure which seems to provide the selective advantage for expansion of erythromycin resistant strains, we pose a hypothesis that the co-purified aPV protect against *ptxP3* strains more efficient, which generated a rare chance for *ptxP3* strains to be under the antibiotic pressure and further developed to be erythromycin resistance.

However, It is a limitation that the cases of *ptxP3* strains were relative too small to give strong evidence about the protection against *ptxP3* lineage by co-purified aPV. Furthermore, the *ptxP3* with the pertactin (PRN) deficient isolates were widely appeared in some industries countries [25], whether the *ptxP3* isolations in this study expressed of PRN were unknown in this study. Lastly, the age of the patients in our study was mainly the infant but not children after at least 5 years of vaccination of co-purified aPV. Thus we can not give powerful support about the protection duration against *ptxP3* linage of the co-purified aPV.

## Conclusions

In conclusion, this study revealed that the erythromycin resistant *B. pertussis* have been steadily increased from 2012 to 2016 in Xi’an, western of China. We also pose a hypothesis that the co-purified aPV containing more antigens has the possibility to protect the infant from being ill of pertussis infected by global popular *ptxP3* lineage *B. pertussis*. To be better understanding the effect of co-purified aPV, an international multicenter cohort study should be performed.

## Data Availability

Xi’an Children’s hospital is the custodian of the data for this study. The data are not accessible online, but may be made available upon written request to the authors, if in line with the Ethical Review Board guidelines.

## Abbreviations

aPV: Acellular pertussis vaccine
ER: Erythromycin resistant
wPV: Whole cell pertussis vaccine
NPs: Nasopharyngeal swabs
OMVs: Outer membrane vesicles
PRN: Pertactin
